# Radiomics for Discriminating Benign and Malignant Salivary Gland Tumors; Which Radiomic Feature Categories and MRI Sequences Should Be Used?

**DOI:** 10.3390/cancers14235804

**Published:** 2022-11-25

**Authors:** Rongli Zhang, Qi Yong H. Ai, Lun M. Wong, Christopher Green, Sahrish Qamar, Tiffany Y. So, Alexander C. Vlantis, Ann D. King

**Affiliations:** 1 Department of Imaging and Interventional Radiology, Faculty of Medicine, Prince of Wales Hospital, The Chinese University of Hong Kong, Hong Kong SAR, China; 2 Department of Health Technology and Informatics, The Hong Kong Polytechnic University, Hong Kong SAR, China; 3 Department of Otorhinolaryngology, Head and Neck Surgery, Prince of Wales Hospital, The Chinese University of Hong Kong, Shatin, New Territories, Hong Kong SAR, China

**Keywords:** radiomics, salivary gland neoplasms, conventional magnetic resonance imaging

## Abstract

**Simple Summary:**

MRI radiomics shows promise in discriminating salivary gland tumors (SGTs) but a consistent radiomics signature has not emerged, partly due to the multitude of initial features fed into the radiomics pipeline. In this study, we investigated the impact of reducing the number of initial radiomic features on the performance of the radiomic models to discriminate between benign and malignant SGTs, by applying six feature categories separately and all feature categories in combination from three anatomical-based MRI sequences. The best models were built by a combination of T1-weighted + logarithm and fat-suppressed T2-weighted + exponential features, which reduced the initial features by 94.0% (from 1015 × 3 to 91 × 2) and achieved an average area under the curve of 0.846. Our results show reducing the number of radiomic features initially analyzed improved feature selection stability without compromising performance. This approach may improve future consensus building on a radiomics signature for discriminating SGTs.

**Abstract:**

The lack of a consistent MRI radiomic signature, partly due to the multitude of initial feature analyses, limits the widespread clinical application of radiomics for the discrimination of salivary gland tumors (SGTs). This study aimed to identify the optimal radiomics feature category and MRI sequence for characterizing SGTs, which could serve as a step towards obtaining a consensus on a radiomics signature. Preliminary radiomics models were built to discriminate malignant SGTs (*n* = 34) from benign SGTs (*n* = 57) on T1-weighted (T1WI), fat-suppressed (FS)-T2WI and contrast-enhanced (CE)-T1WI images using six feature categories. The discrimination performances of these preliminary models were evaluated using 5-fold-cross-validation with 100 repetitions and the area under the receiver operating characteristic curve (AUC). The differences between models’ performances were identified using one-way ANOVA. Results show that the best feature categories were logarithm for T1WI and CE-T1WI and exponential for FS-T2WI, with AUCs of 0.828, 0.754 and 0.819, respectively. These AUCs were higher than the AUCs obtained using all feature categories combined, which were 0.750, 0.707 and 0.774, respectively (*p* < 0.001). The highest AUC (0.846) was obtained using a combination of T1WI + logarithm and FS-T2WI + exponential features, which reduced the initial features by 94.0% (from 1015 × 3 to 91 × 2). CE-T1WI did not improve performance. Using one feature category rather than all feature categories combined reduced the number of initial features without compromising radiomic performance.

## 1. Introduction

Salivary gland tumors (SGTs) account for 2–6.5% of all head and neck tumors [1,2]. Around 80% of SGTs arise from the parotid gland of which about 80% are benign (BSGT), mainly pleomorphic adenoma (PA) and Warthin’s tumor (WT) while the remaining 15–30% are malignant (MSGTs) [3,4,5], mainly comprising mucoepidermoid carcinoma, acinic cell carcinoma, adenoid cystic carcinoma, carcinoma ex-pleomorphic adenoma, and adenocarcinoma [6]. Magnetic resonance imaging (MRI) is often the preferred imaging modality in patients with a histologically proven SGT, to map the extent of disease and image deep surrounding structures with advantages over CT for the avoidance of ionizing radiation, better contrast resolution especially in the depiction of local spread including peri-neural disease, and visualization of the relationship of the tumor to the facial nerve and branches [5,7]. However, there are situations when the pathology of the SGT is unknown, such as incidentally found SGTs on a head and neck MRI examination or when tumor location limits access to biopsy or fine needle aspiration cytology. Furthermore, malignant SGTs may be missed or over-diagnosed using parotid fine-needle aspiration cytology and there may be sampling difficulties related to SGT heterogeneity [8,9].

Morphological features of MSGTs on MRI include irregular margins, invasion beyond the gland into adjacent structures, perineural extension, and metastatic regional lymph nodes. Signal intensity and markers from functional MRI techniques such as diffusion-weighted imaging and dynamic contrast-enhanced MRI also help to discriminate MSGTs and BSGTs [9,10]. However, there is still overlap in appearances, notably some benign tumors have irregular margins while low grade malignancies may have benign features [11,12,13]. Moreover, MRI-based tumor diagnosis by visual qualitative assessment relies primarily on the radiologist’s experience, which could lead to objective variations, especially in the complex head and neck region [14,15]. Therefore, quantitative methods to characterize SGTs on MRI could facilitate the clinical workflow by improving diagnostic accuracy and reducing inter-observer variability.

Radiomic analysis involves high-throughput quantitative imaging features extracted from a region of interest in medical images [16]. The radiomic signature produced by combining the best-performing features could be used to discriminate between different tumor types. Such radiomic signatures from MRI have shown early promise in discriminating SGTs [17,18,19,20,21,22,23]. Nonetheless, in keeping with most other radiomics oncology studies, a consistent MRI radiomic signature has not emerged in the literature, limiting the widespread clinical application of radiomics for the discrimination of SGTs.

Radiomic packages commonly involve thousands of features, comprising six main feature categories: shape, first-order features, texture features as well as filtered base derivatives (i.e., exponential, logarithm and wavelet) [24]. These numerous features are commonly extracted from relatively small-sized MRI samples, which causes dimensionality problems [25,26,27] that must be tackled by additional methods and steps such as least absolute shrinkage and selection operator (LASSO), ridge and elastic net, …, etc. to shrink the dimensionality and identify potential features [28,29,30]. However, when the initial pool of features is very large, it is unlikely that these techniques will select the same combination of features every time to produce a stable radiomics model [28,29,30,31,32,33]. With so many features to evaluate at the start of the analysis, it is not surprising that radiomic signatures for discriminating SGTs rarely contain the same combination of features which limits the wider use of radiomics in clinical practice. To take a step towards improving stability of the selected radiomic signature, it would be advantageous to limit the number of initial feature categories fed into the radiomic pipeline while still providing acceptable diagnostic performance.

The aim of this study was to determine if we could improve feature selection stability by restricting the initial number of radiomic features fed into the pipeline without com-promising the performance of MRI radiomic features in discriminating between BSGTs and MSGTs. We evaluated the impact of restricting the number of initial features by narrowing down to one feature category and MRI sequences, comparing between T1-weighted (T1WI), fat-suppressed T2WI (FS-T2WI) and contrast-enhanced T1WI (CE-T1WI) images.

## 2. Materials and Methods

### 2.1. Patient Characteristics

This retrospective study was approved by our Institutional Review Board, and the requirement for informed consent was waived. The enrolled patients had histologically confirmed SGTs (34 MSGTs, 57 BSGTs) and had undergone three MR examinations that included axial T1-weighted (T1WI), fat-suppressed T2WI (FS-T2WI) and contrast-enhanced T1WI (CE-T1WI) images. The patient demographics and tumor distributions are detailed in Table 1.

### 2.2. Image Acquisition

All MRI examinations were performed on a Philips 3.0 Tesla scanner (Achieva TX, Phillips Healthcare, Best, The Netherlands) with a 16-channel head and neck coil for radiofrequency pulse transmission and a neurovascular phased-array coil for imaging reception. The data acquisition protocols consisted of axial (1) T1WI: repetition time (TR) = 298–715 ms, echo time (TE) = 10 ms, echo number = 1, slice thickness = 4 mm, flip angle = 90°; (2) FS-T2WI: TR = 1825–5412 ms, TE = 80 ms, slice thickness = 4 mm, fat-suppression technique and spectral attenuation inversion recovery; and (3) CE-T1WI: TR = 298–655 ms, TE = 10 ms, echo number = 1, slice thickness = 4 mm, flip angle = 90°.

### 2.3. Tumor Segmentation

All salivary gland tumors on each MRI sequence were manually segmented by a researcher (Q.Y.H.A.) with seven years of experience with MRI of head and neck tumors, using open-source software ITK-SNAP (version 3.4.0; http://www.itksnap.org, accessed on 1 October 2020) [34]. To assess inter-observer agreement, 30 salivary gland tumors (10 MTs, 10 PAs and 10 WTs) were randomly selected and manually segmented on each MRI sequence by a second researcher (S.Q.) with four years of experience with head and neck MRI who was blind to the patients’ diagnoses and the segmentation of the first researcher (Q.Y.H.A.). The volumetric dice similarity coefficient (DSC) [35] was used to calculate the inter-observer segmentation agreement. DSC of <0.6, 0.6–<0.8, 0.8–1.0 and 1.0 indicates inadequate, good, very good and ideal consistency, respectively [36].

### 2.4. Image Pre-Processing

To normalize image intensity and minimize the effects of variation in the weighted MRI scanning parameters, the N4ITK bias field correction algorithm was implemented to remove the artifacts caused by the inhomogeneity of the scanner’s magnetic field [37]. The well-established “µ ± 3σ” algorithm was applied to identify and remove image intensity outliers [38]. Next, the images were resampled to the median spacing of the training cases. Lastly, z-score normalization was implemented in the non-zero areas for intensity normalization [39].

### 2.5. Feature Extraction

From the volume of interest of the segmented tumors on each MRI sequence (T1WI, CE-T1WI and FS-T2WI), 1015 3D quantitative features were extracted using PyRadiomics (version 3.0.1) (available at https://pyradiomics.readthedocs.io/en/latest/, accessed on 5 November 2020) [24]. All quantitative features (*n* = 1015) were divided into six feature categories: (1) shape (*n* = 14), (2) first-order (*n* = 18), (3) texture (*n* = 73), (4) exponential (*n* = 91), (5) logarithm (*n*= 91) and (6) wavelet (*n* = 801) (Datasheet S1). Image pre-processing and feature extraction operation was performed using the open-source packages SimpleITK (version 2.1.1) [40] and PyRadiomics (version 3.0.1) [24] on Python (version 3.7.10) programing language. 

### 2.6. Data Augmentation

To reduce potential bias caused by imbalance between the number of positive and negative samples, data augmentation was performed using a synthetic minority oversampling technique (SMOTE) [41,42] in both the training and validation sets. Data augmentation was performed on the MATLAB 2020a (MathWorks, Natick, MA, USA) software platform, using an open-source package available at https://github.com/Nekooeimehr/MATLAB-Source-Code-Oversampling-Methods, accessed on 5 November 2020).

### 2.7. Feature Selection

All features and features grouped by the six feature categories extracted from T1WI, CE-T1WI and FS-T2WI were pooled for the feature selection procedure. Before feature selection, quantitative features were standardized by z-score normalization. Radiomic feature selection was performed on the training dataset. For each cross-validation loop, first, the *p*-values of the individual features of the internal training set were calculated using the two-tailed unpaired *t*-test (for features with normalized distribution) and the Wilcoxon rank-sum test (for features with non-normalized distribution) [43], features with a *p* < 0.05 were enrolled for the next step; second, the LASSO algorithm [44], a well-established dimensionality shrinkage approach, was performed to identify the potential features with the regularization parameter (λ) determined using the minimum criteria via 10-fold cross-validation; third, the features with non-zero LASSO coefficients were registered as the selected features.

### 2.8. Radiomics Models Construction and Evaluation

**Figure 1:**
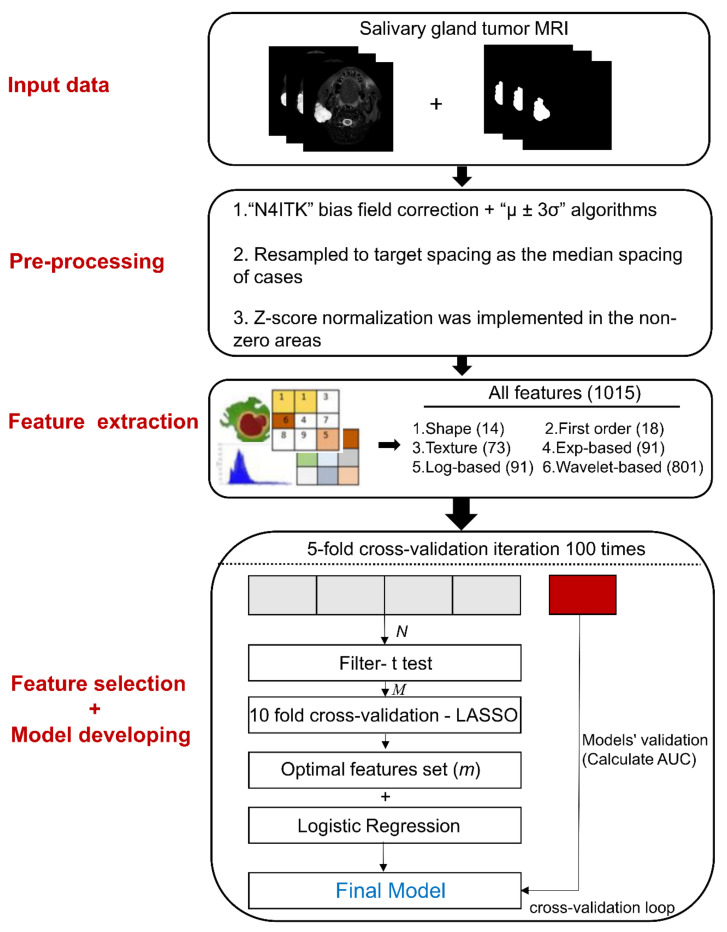
Overview of the radiomic analysis framework to differentiate benign and malignant salivary gland tumors. Region of interest segmentation was performed by experienced clinical researchers. After pre-processing, radiomic features were extracted and categorized into six groups. Feature selection was performed using 10-fold cross-validation in the training dataset. Radiomic models were constructed using multivariable logistic regression classifiers for salivary gland tumor differentiation. Finally, models were validated by 5-fold cross-validation with 100 repetitions. Exp = exponential; Log = logarithm; LASSO = least absolute shrinkage and selection operator; AUC = the area under the receiver operating characteristic curve.

A clinically recognized multivariable logistic regression (LR) classifier was used to construct radiomic models with the selected features. We applied 5-fold cross-validation with 100 repetitions to assess model performance. The performance metric was the area under the receiver operating characteristic curve (AUC). The stability strength of the feature selection for the model building was evaluated using Nogueira score [29,31] and Jaccard index [31]. The feature selection, model construction and evaluation were performed using in-house code and the “Glmnet” package [45] (Qian, J. http://www.stanford.edu/~hastie/glmnet_matlab/, accessed on 5 November 2020) on the MATLAB R2020a (MathWorks, Natick, MA, USA). The schematic workflow of the radiomics method is shown in Figure 1.

### 2.9. Selection of the Best Sequences and Feature Categories

The performances (AUCs) of the models based on (1) shape (*n* = 14), (2) first-order (*n* = 18), (3) texture (*n* = 73), (4) exponential (*n* = 91), (5) logarithm (*n* = 91), (6) wavelet (*n* = 801) and all features (*n* = 1015) for each sequence (T1WI, CE-T1WI and FS-T2WI) were compared to determine the best feature category for each sequence. Next, to investigate whether the various combinations of sequences could improve performance, radiomics models were built by adding sequences one by one according to the performance of each sequence.

### 2.10. Statistical Analysis

The AUCs of all models were compared using a one-way analysis of variance (ANOVA). Differences between the age, sex of the patients and Jaccard index of the different methods were compared using an independent samples *t*-test. The difference between the Nogueira score of the different methods was tested using a technique reported by S. Nogueira [29]. In all conditions, a *p* < 0.05 was considered statistically significant. Statistical analysis was performed using GraphPad Prism software (version 5.01, Dotmatics, San Diego, CA, USA).

## 3. Results

### 3.1. Radiomic Analysis to Discriminate between MSGTs and BSGTs

In the training set, all sequences with the corresponding feature categories and all feature categories combined (except for the first-order features, where the AUC was 0.554 for T1WI and 0.625 for CE-T1WI) showed the potential to discriminate between MSGTs and BSGTs, with AUCs of T1WI: 0.721–0.999, FS-T2WI: 0.833–0.998 and CE-T1WI: 0.706–0.997. Similar to the training set, all sequences in the validation set, other than the first-order features (AUCs of 0.552 for T1WI and 0.605 for CE-T1WI), showed the potential to discriminate between MSGTs and BSGTs, with AUCs of T1WI: 0.718–0.828, FS-T2WI: 0.774–0.819 and CE-T1WI: 0.689–0.754 (Table 2).

### 3.2. Performance Comparison of Each Feature Category and All Features Combined

To discriminate between MSGTs and BSGTs on each MRI sequence, the following feature categories with the best discriminate performances were identified according to the validation results shown in Table 2. For T1WI, the logarithm-based features (AUC of 0.828) were compared with other feature subcategories (AUCs of 0.552–0.801, *p* < 0.001). For FS-T2WI, the exponential-based features (AUC of 0.819) were compared with other feature subcategories (AUCs of 0.778–0.806, *p* < 0.001). For CE-T1WI, the logarithm-based features (AUC of 0.754) were compared with other feature subcategories (AUCs of 0.605–0.747, *p* < 0.001). For all sequences, preliminary results built using the best performing feature category achieved significantly higher AUCs than those built using all features combined: T1WI, 0.828 vs. 0.750; FS-T2WI, 0.819 vs. 0.774; and CE-T1WI, 0.754 vs. 0.707, respectively (*p* < 0.001).

### 3.3. Comparison of Stability Strength and Number of Features Based on the Best Features Category and All Combined Features

The feature selection stability based on the best features category achieved higher values than all features combined both in Nogueira score (T1WI: 0.437 vs. 0.360, FS-T2WI: 0.466 vs. 0.292, CE-T1WI: 0.433 vs. 0.331) and Jaccard index (T1WI: 0.330 vs.0.234, FS-T2WI: 0.368 vs.0.184, CE-T1WI: 0.322 vs.0.219) (all *p* < 0.001, Table 3). Using the best feature category (logarithm for T1WI and CE-T1WI, *n* = 91; exponential for FS-T2WI, *n* = 91) for each sequence and for T1WI and FS-T2WI combined reduced the initial input of features from 1015 to 91 (91.0%) and from 1015 × 3 to 91 × 2 (94.0%), respectively.

### 3.4. Selection of MRI Sequences to Discriminate between MSGTs and BSGTs

Using the best feature category for each sequence, the preliminary radiomics models built on T1WI-logarithm, T1WI-logarithm combined with FS-T2WI-exponential and T1WI-logarithm combined with FS-T2WI-exponential and CE-T1WI-logarithm achieved AUCs of 0.828, 0.846 and 0.825; accuracies of 0.750, 0.761 and 0.751; sensitivities of 0.730, 0.740 and 0.728; and specificities of 0.769, 0.782 and 0.775, respectively, in validation set (Table 4).

### 3.5. Inter-Observer Agreement for Segmentation

The inter-observer agreement for tumor segmentation on T1WI, FS-T2WI and CE-T1WI showed mean DSC values of 0.843 ± 0.065, 0.862 ± 0.059 and 0.827 ± 0.067, respectively (Datasheet S2).

### 3.6. Additional Analysis to Further Reduce Radiomic Features

Having completed the aim of this study and shown that reducing from the number of initial features (1015 × 3 to 91 × 2) fed into the radiomics pipeline (restricting feature categories and MRI sequences) improved feature selection stability without compromising performance, we then analyze here a second step to further reduce the number of features that is essential for building a final radiomic model (Supplementary Text S1).

## 4. Discussion

In this study, we investigated the performance of radiomics based on conventional MRI sequences in discriminating between MSGTs and BSGTs. The aim was to determine if the diagnostic performance of MRI radiomic features would be reduced by restricting the initial number of radiomic features fed into the pipeline. We evaluated the impact of reducing features by restricting initial feature categories and MRI sequences.

We found that all radiomic categories for all three sequences, except for first-order features extracted from T1WI and CE-T1WI, could distinguish MSGTs from BSGTs. The diagnostic performance was not affected by restricting analysis to one feature category rather than combining all feature categories. We also showed that feature extraction could be reduced by confining extraction to only two of the three MRI sequences because the CE-T1WI did not outperform either the T1WI or FS-T2WI sequences (Table 2). The best overall performance was obtained by combining T1WI and FS-T2WI to produce an AUC of 0.846, with an accuracy of 0.761, a sensitivity of 0.740 and a specificity of 0.782 (Table 4).

The exponential features were the best categories for FS-T2WI (AUC of 0.819), and the logarithm features were best for T1WI (AUC of 0.828) and CE-T1WI (AUC of 0.754). Although feeding more features into the radiomics pipeline improved the radiomic model performance in the training set, a wider choice of initial features decreased the feature selection stability and model performance in the validation set. Specifically, the feature categories with a smaller number of features (shape and first-order, *n* = 14–18) showed similar results in both the training and validation sets; feature categories with an intermediate number of features (texture, exponential and logarithm, *n* = 73–91) showed a slight drop in performance in the validation sets; and feature categories with the largest number of features (wavelet and all categories combined, *n*= 801–1015) showed the largest drop in performance such that these categories no longer had the best performance (Table 2). This finding was also supported by the stability strength of the best performing feature category in all three MRI sequences. Specifically, reducing the initial input features to the best feature categories to build a radiomic model could also improve the stability strength (Table 3).

Our results indicate that including more radiomic features and categories in analysis might not produce a better model. It has been observed in practice that if the amount of training data used is small compared to the number of features, adding too many features degrades the metrics of the classifier [46]. High dimensionality and a small sample size increase the overfitting risk and decrease the classifiers accuracy, posing challenges to classification techniques [32,33]. Moreover, as classifiers rarely scale well to vast numbers of features, high dimensionality can lead to unreasonably long computation time [33]. Thus, analyzing thousands of radiomic features in a small group of samples usually restricts the use of radiomics in clinical practice [47,48]. Limiting the number of features in the initial pool could overcome some of these problems without compromising diagnostic accuracy.

The categorized features investigated in this study could provide more valuable and efficient potential markers for discriminating SGTs based on radiomic analysis. As there can be large overlaps in the shapes and sizes of malignant and benign tumors, it is not surprising that this category did not perform well. We cannot explain why the logarithm and exponential features performed best. However, studies of other MRI sequences, such as diffusion-weighted imaging (DWI) [49,50], have shown that malignant tumors may have features that fall between those of PA and WT which are the two most common benign tumors. It is possible, therefore, that logarithm and exponential, which are both filtered features, were able to change the distribution of features between these three groups so that there is a greater difference between BSGTs and MSGTs.

The study results indicate that numerous features included in radiomic packages are unnecessary and inefficient as initial input features to construct a radiomics model. Both previous studies and our results indicate shape and first-order features are not primary choices for radiomic analysis based on conventional MRI sequences to discriminate between MSGTs and BSGTs [20,51]. Moreover, although several studies show radiomics features could discriminate SGTs [19,20,22,51] but there is little overlap of selected features and no consensus on a radiomic signature. Zheng YM [20] selected 15 wavelets, one texture and one first-order feature for the final radiomic signature to discriminate benign from malignant tumors. Not surprisingly, the wavelets then dominated the radiomic signature, given such a number of wavelet features (1488/1702) being fed into the radiomics procedure. Three other studies [19,22,51] which proposed a radiomic analysis also lacked consensus on the selection of features. At present, most discussion about radiomics variability has focused on image scanner instrumentation manufacturers, parameter settings, tumor segmentation, pre-processing methods and radiomic software packages [38,52,53,54,55,56,57], but little attention has been paid to the problem of variability caused by the abundance of initial features.

This study reduced dimensionality by more than 90% simply by using a single feature category to build radiomics models, and then demonstrated its the effectiveness and potential for radiomics research without reducing performance. Surprisingly, there is little data in the literature on the value of radiomics analysis of the T1WI contrast image. In radiomics, the discrimination of SGTs have mostly reported results from T1WI, T2WI and DWI [18,19,20,21]. Only three machine learning study [58,59,60], using deep learning instead of radiomics, evaluated CE-T1WI images of the parotid gland. In agreement with our study, one of them found that CE-T1WI did not improve the classification of SGTs [59]. It should be emphasized that while contrast enhanced images may not provide additional valuable radiomic features for SGT discrimination they are still a necessity for clinical evaluation of SGTs, especially for documenting extent of spread of MSGTs. The aim of this study was to take the first step in reducing the number of evaluated features in order to facilitate reaching a consensus on the radiomic signature. We have substantially decreased the number of features from 1015 × 3 to 91 × 2. However, we believe it will require a second step to further reduce the number of features before model building. We do not know the best method for this second step and believe it is an important area for future research, but we have proposed a at present have included a possible direction in the supplementary material that by using repetitive cross-validation to optimize the trade-off between feature stability and model performance, and reduces the features to four, results shown in the supplementary material (Supplementary Text S1). This method takes advantage of resampling to identify features that are not data-specific and, hence, have lower risk of overfitting.

This study has some limitations. First, the study lacks external validation. Nonetheless, the performance of radiomic analysis for discriminating MSGTs and BSGTs was verified by cross-validation. Second, as the focus of this study was conventional anatomical MRI imaging, we did not evaluate other MRI sequences such as DWI, which has yielded variable results and no consensus on the best radiomic signature [18,21]. Third, sampling bias may exist because of the small sample size in our study.

## 5. Conclusions

Reducing the number of features fed into the radiomics pipeline could help researchers take the first step towards achieving a consensus on a radiomic signature for discriminating between BSGTs and MSGTs. In this study we found feature selection stability was improved without compromising performance by restricting the number of initial MRI sequences analyzed to T1WI and FS-T2WI images (avoiding the contrast enhanced T1W images) and restricting the number of initial feature categories to one category per sequence (logarithm for T1WI, and exponential for FS-T2WI) rather than using all feature categories combined. We hope that future studies will evaluate the role of feature category and MRI sequence selection to determine the strongest candidates for improving feature selection stability to facilitate reaching a radiomic signature consensus.

## Figures and Tables

**Table 1:** General characteristics of the patient cohort. Two-tailed unpaired student *t*-test was used to evaluate differences in characteristics across MSGTs and BSGTs.

Characteristics	MSGT (*n* = 34)	BSGT (*n* = 57)	*p*-Value
Tumor histology	Lymphoepithelioma-like carcinoma 7 (20.6%) Myoepithelial carcinoma 2 (5.9%) Salivary duct carcinoma 4 (11.8%) Adenoid cystic carcinoma 5 (14.7%) Mucoepidermoid carcinoma 8 (21.6%) Metastatic carcinoma 2 (5.9%) Acinic cell carcinoma 1 (2.9%) Poorly differentiated carcinoma 2 (5.9%) Basal cell adenocarcinoma 1 (2.9%) Other carcinomas 2 (5.9%)	Pleomorphic adenoma 44 (77.2%) Warthin’s tumor 13 (22.8%)	
Sex (M/F)	20/14	30/27	0.57
Age (years)	57.94 ± 16.76	55.35 ± 15.91	0.25
Tumor location	Parotid 25 (73.5%) Submandibular 5 (14.7%) Sublingual 4 (11.8%)	Parotid 51 (89.5%) Submandibular 6 (10.5%) Sublingual 0 (0%)	
Tumor site	Unilateral 34 (100%) Bilateral 0 (0%)	Unilateral 47 (90.4%) Bilateral 5 (9.6%)	

Numerical data are presented as means ± standard deviations, categorical data as numbers (*n*). M = male, F = female; MSGT = malignant salivary gland tumor; BSGT = benign salivary gland tumor.

**Table 2:** The performance (AUC) of MRI sequence and feature category for discriminating MSGTs from BSGTs.

	Shape (*n* = 14)	First Order (*n* = 18)	Texture (*n* = 73)	Exp (*n* = 91)	Log (*n* = 91)	Wavelet (*n* = 801)	All Features (*n* = 1015)
Validation set							
T1WI	0.718 ± 0.004	0.552 ± 0.003	0.801 ± 0.004	0.729 ± 0.004	0.828 ± 0.004 ***	0.725 ± 0.005	0.750 ± 0.004
FS-T2WI	0.778 ± 0.004	0.788 ± 0.004	0.785 ± 0.004	0.819 ± 0.004 ***	0.806 ± 0.004	0.785 ± 0.005	0.774 ± 0.004
CE-T1WI	0.704 ± 0.004	0.605 ± 0.003	0.729 ± 0.004	0.747 ± 0.004	0.754 ± 0.005 ***	0.689 ± 0.004	0.707 ± 0.004
Training set							
T1WI	0.721 ± 0.003	0.554 ± 0.003	0.871 ± 0.003	0.835 ± 0.002	0.902 ± 0.001	0.996 ± 0.000	0.999 ± 0.000
FS-T2WI	0.833 ± 0.002	0.841 ± 0.001	0.891 ± 0.001	0.924 ± 0.001	0.926 ± 0.001	0.998 ± 0.000	0.998 ± 0.000
CE-T1WI	0.706 ± 0.002	0.625 ± 0.004	0.845 ± 0.002	0.862 ± 0.001	0.866 ± 0.002	0.980 ± 0.001	0.997 ± 0.000

Numerical data are presented as means ± standard errors. AUC = area under the receiver operating characteristic curve; MSGT = malignant salivary gland tumor; BSGT = benign salivary gland tumor; *n* = the initial feature number entered the radiomics procedure; T1WI = T1-weighted imaging; FS-T2WI = fat-suppressed T2-weighted imaging; CE-T1WI = contrast-enhanced T1WI. Exp = exponential; log = logarithm; differences were considered statistically significant for *** = *p* < 0.001. Bold indicates the highest AUC mean value for each sequence in the validation set.

**Table 3:** Stability strength of feature selection based on different methods.

	All Features	Best Feature Category	*p*-Values
Nogueira score			
T1WI	0.360	**0.437**	<0.001
FS-T2WI	0.292	**0.466**	<0.001
CE-T1WI	0.331	**0.433**	<0.001
Jaccard index			
T1WI	0.234 ± 0.066	**0.330 ± 0.145**	<0.001
FS-T2WI	0.184 ± 0.069	**0.368 ± 0.150**	<0.001
CE-T1WI	0.219 ± 0.067	**0.322 ± 0.137**	<0.001

Numerical data are presented as means ± standard deviation. T1WI = T1-weighted imaging; FS-T2WI = fat-suppressed T2-weighted imaging; CE-T1WI = contrast-enhanced T1WI. Differences were considered statistically significant for *p* < 0.05. Bold indicates the highest Nogueira score and Jaccard index for each sequence.

**Table 4:** Assessing the performances of the combined MRI sequence based on the best feature category to discriminate MSGTs from BSGTs.

	Validation Set				Training Set	
	T1WI-Log (*n* = 91)	T1WI-Log + FS-T2WI-Exp (*n* = 182)	T1WI-Log + FS-T2WI-Exp + CE-T1WI-Log (*n* = 273)	T1WI-Log (*n* = 91)	T1WI-Log + FS-T2WI-Exp (*n* = 182)	T1WI-Log + FS-T2WI-Exp + CE-T1WI-Log (*n* = 273)
AUC	0.828 ± 0.004	0.846 ± 0.004 **	0.825 ± 0.005	0.902 ± 0.001	0.953 ± 0.001	0.978 ± 0.001
Accuracy	0.750 ± 0.004	**0.761 ± 0.004**	0.751 ± 0.004	0.837 ± 0.002	0.885 ± 0.001	0.951 ± 0.001
Sensitivity	0.730 ± 0.005	**0.740 ± 0.005**	0.728 ± 0.005	0.846 ± 0.002	0.893 ± 0.002	0.950 ± 0.001
Specificity	0.769 ± 0.005	**0.782 ± 0.005**	0.775 ± 0.005	0.826 ± 0.003	0.878 ± 0.002	0.951 ± 0.001

Numerical data are presented as means ± standard errors. MSGT = malignant salivary gland tumor; BSGT = benign salivary gland tumor; *n* = the initial feature number entered the radiomics procedure; T1WI = T1-weighted imaging; FS-T2WI = fat-suppressed T2-weighted imaging; CE-T1WI = contrast-enhanced T1WI; exp = exponential; log = logarithm; differences were considered statistically significant for **: 0.001 < *p* < 0.01. Bold indicates the best performance in the validation set.

## Data Availability

Data and clinical information presented in this study can be provided upon requests from the corresponding authors.

## Supplementary Materials

The following supporting information can be downloaded at: https://www.mdpi.com/article/10.3390/cancers14235804/s1, Datasheet S1: The name list of 1015 3D quantitative features and six feature categories: extracted in this study; Datasheet S2: The volumetric dice similarity coefficient for the 30 randomly selected inter-observer segmentations on T1WI, FS-T2WI and CE-T1WI. Text S1: Additional analysis to further reduce radiomic features.
