# Supplementary material for: Radiomics for Discriminating Benign and Malignant Salivary Gland Tumors; Which Radiomic Feature Categories and MRI Sequences Should Be Used?
Source: Cancers (Basel). 2022 Nov 25;14(23):5804. doi: 10.3390/cancers14235804 (PMC9740105; doi:10.3390/cancers14235804)
Supplement: Supplementary file 1 [file cancers-14-05804-s001.zip › Text S1.pdf]

Supplementary Text S1 –Additional analysis to further reduce radiomic features

1. Method

The top ten radiomic features were selected based on the highest frequency from the best combination of MRI sequences and feature categories, as reported in the main study i.e., T1WI-log + FS-T2WI-exp. The frequency, which reflects feature stability, was defined as the number of times a feature was selected by the LASSO method in the training set based on the 5-fold cross-validation with 100 repetitions. To find the optimal feature set with the highest performance and the minimum number of features, we evaluated radiomic models constructed from an increasing number of features (features were added one at a time in sequential order based on selection frequency up to the maximum of ten features). For each of the ten feature sets, the multivariable LR classifier was used for model building. The highest AUC values averaged across the five folds validation set were used to determine the optimal number of features to be included in the final radiomic model to discriminate MSGTs from BSGTs.

The final model is the weighted-average ensemble of the five models corresponding to each of the five folds in the cross-validation. Its performance was estimated by averaging across the five-fold cross-validation to obtain AUC, accuracy, sensitivity, and specificity in discriminating MSGTs and BSGTs.

2. Results

The ten features with the highest selection frequency are shown in Figure S1.A. The optimal number of features was four as determined by the flattening of AUC in the training set (Figure S1.B) and the drop in the AUC in the validation set (Figure S1.C). The fitted coefficients, intercepts and cut-off values and their weighted sums of each radiomics LR classifier built on each fold using the four optimal radiomic features are shown in Table S1. This radiomic model achieved a mean AUC of 0.927 and 0.917, accuracy of 0.868 and 0.846, sensitivity of 0.861 and 0.852, and specificity of 0.873 and 0.841 in the training set and validation set, respectively, to discriminate between MSGTs and BSGTs (Table S2). The ROC curves of each fold of the final model for the training set and validation set are shown in Figure S2. A and Figure S2.B.

The results suggested that using initial features from the best feature category and MRI sequences determined in the main study, only four features, in contrast to >16 features reported in the literature[20,22], were enough to build a radiomic model with good performance for discrimination of MSGTs and BSGTs.

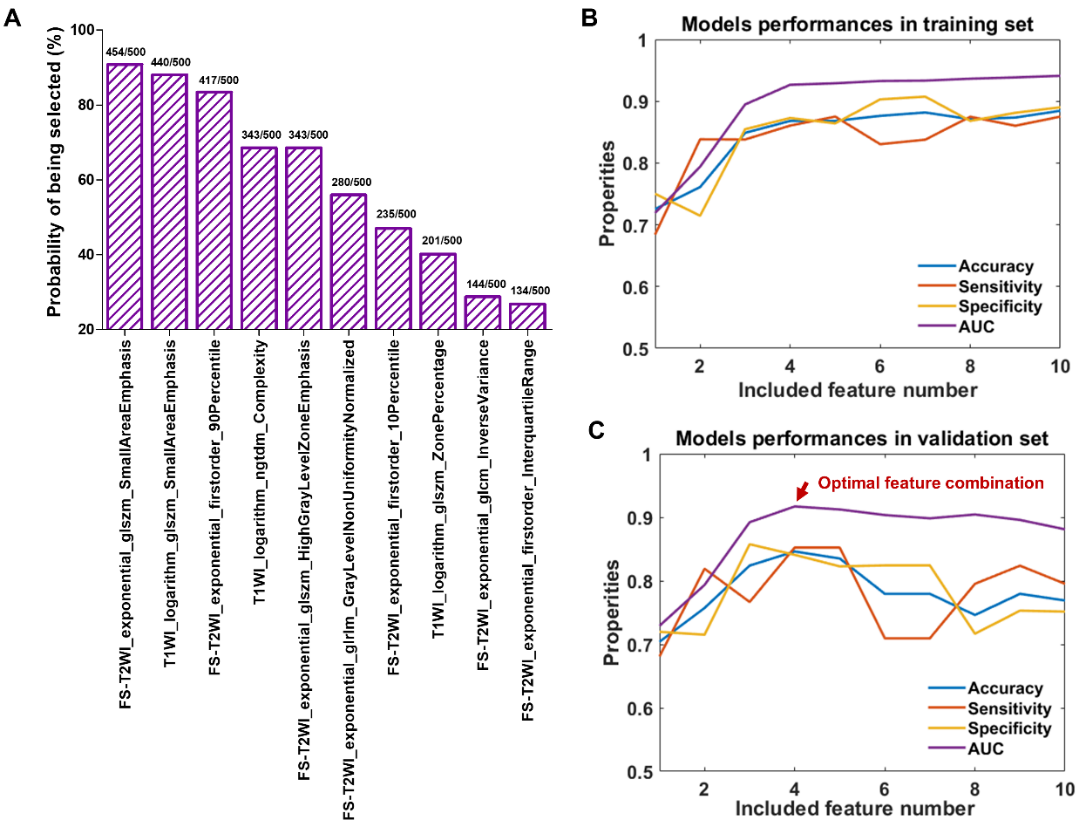

Figure S1. (A) The top ten features ordered by the highest frequency of selection in the training set from T1WI-log + FS-T2WI-exp feature category. (B) Performance of the radiomic models with accumulating number of features in the training set and (C) in the validation set. T1WI = T1-weighted imaging; FS-T2WI = fat-suppressed T2-weighted imaging; exp = exponential; log = logarithm; AUC = the area under the receiver operating characteristic curve.

Table S1. Features in the optimal feature set selected from T1WI-log + FS-T2WI-exp feature category. The coefficients and their weighted average for the final ensemble model of each feature in the radiomics LR models were constructed in 5 folds to discriminate MSGTs from BSGTs.

Sequences	Feature name	Fold1 (LR1)	Fold2 (LR2)	Fold3 (LR 3)	Fold4 (LR4)	Fold5 (LR5)	Ensemble (LR)
FS-T2WI	exponential_glszm_SmallAreaEmphasis	-1.110	-0.537	-0.479	-0.371	-0.335	-0.5134
T1WI	logarithm_glszm_SmallAreaEmphasis	-1.618	-1.025	-1.482	-1.093	-1.275	-1.1880
FS-T2WI	exponential_firstorder_90Percentile	-1.769	-2.327	-2.426	-2.104	-1.927	-1.9399
T1WI	logarithm_ngtdm_Complexity	-1.711	-1.678	-1.857	-2.404	-1.808	-1.7329
Intercepts		-1.553	-1.777	-1.590	-2.001	-1.599	-1.5625

MSGT = malignant salivary gland tumor; BSGT = benign salivary gland tumor; T1WI = T1-weighted imaging; FS-T2WI = fat-suppressed T2-weighted imaging; exp = exponential; log = logarithm; LR = logistic regression; glszm = gray-level = gray-level size zone matrix; ngtdm = neighboring gray-tone difference matrix.

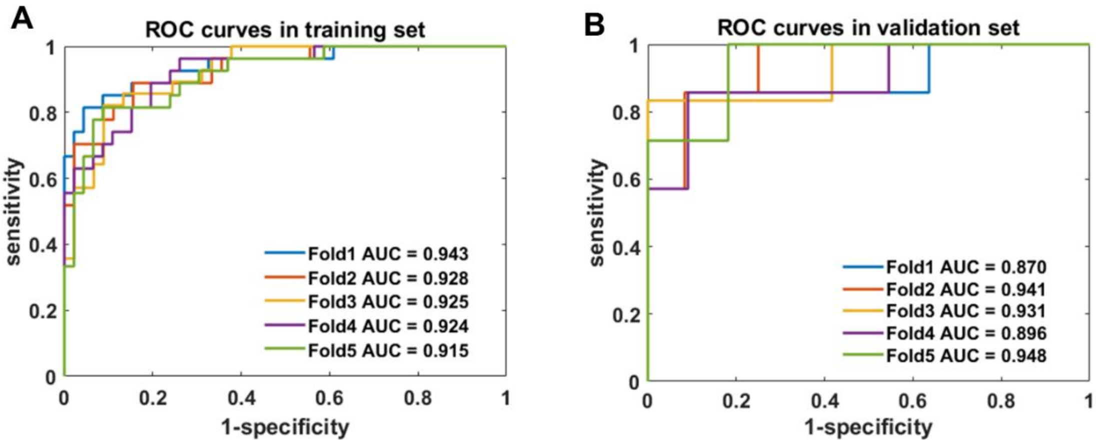

Figure S2. The ROC curves of the radiomic models constructed from the 4 optimal features in (A) training set and (B) validation set for discriminating MSGTs from BSGTs. ROC = receiver operating characteristic curve; MSGT = malignant salivary gland tumor; BSGT = benign salivary gland tumor; AUC = the area under the receiver operating characteristic curve.

Table S2. Performance of radiomics model constructed based on the optimal feature set selected from T1WI-log + FS-T2WI-exp feature category for discriminating between MSGTs and BSGTs averaged across the five folds.

	Training set	Validation Set
AUC	0.923 ± 0.005	0.917 ± 0.015
Accuracy	0.868 ± 0.014	0.846 ± 0.011
Sensitivity	0.861 ± 0.029	0.852 ± 0.045
Specificity	0.873 ± 0.038	0.841 ± 0.035

Numerical data are presented as means ± standard errors. MSGT = malignant salivary gland tumor; BSGT = benign salivary gland tumor; T1WI = T1-weighted imaging; FS-T2WI = fat-suppressed T2-weighted imaging; exp = exponential; log = logarithm; AUC = the area under the receiver operating characteristic curve.
